# NEURL rs6584555 and CAND2 rs4642101 contribute to postoperative atrial fibrillation: a prospective study among Chinese population

**DOI:** 10.18632/oncotarget.9422

**Published:** 2016-05-17

**Authors:** Tiemin Wei, Jingjing Song, Min Xu, Lingchun Lv, Chong Liu, Jiayi Shen, Ying Huang

**Affiliations:** ^1^ Vasculocardiology Department, The Fifth Affiliated Hospital of Wenzhou Medical University, Affiliated Lishui Hospital of Zhejiang University, The Central Hospital of Zhejiang Lishui, Lishui, Zhejiang, P.R. China; ^2^ Department of Radiology, The Fifth Affiliated Hospital of Wenzhou Medical University, Affiliated Lishui Hospital of Zhejiang University, The Central Hospital of Zhejiang Lishui, Lishui, Zhejiang, P.R. China; ^3^ Department of Cardiology, Sichuan Medical University, Sichuan, P.R. China

**Keywords:** susceptibility, atrial fibrillation, coronary artery bypass graft surgery, SNP

## Abstract

Postoperative atrial fibrillation (POAF) is a serious, common complication after coronary artery bypass grafting (CABG) surgery. Recently, 5 novel loci were identified to be associated with atrial fibrillation susceptibility using a combination of genotyping, eQTL mapping, and functional validation. In current study, we aim to evaluated the positive findings for POAF susceptibility after CABG among Chinese population, using a population-based, two-stage, nested case-control study with 1,400 patients. NEURL rs12415501 and CAND2 rs4642101 were significantly associated with POAF susceptibility after CABG among Chinese population in both stages. When pooled together, the ORs for each additional copy of minor allele was 1.29 (95% CI: 1.13-1.48, *P* = 1.7×10^−4^) for NEURL rs12415501, and 1.21 (95% CI: 1.08-1.36, *P* = 9.8×10^−4^) for CAND2 rs4642101. Functional validation experiments found the AF risk allele of NEURL rs6584555 and CAND2 rs4642101 correlated with an increased expression of its corresponding genes (*P*<0.001). In this independently collected cardiac surgery cohort, we replicated the previous findings, and 2 novel loci are independently associated with POAF risk in patients who undergo CABG surgery in Chinese population.

## INTRODUCTION

Postoperative atrial fibrillation (POAF) is a serious yet common complication, also a known predictor of in-hospital morbidity and short-term survival after coronary artery bypass grafting (CABG) surgery, occurring in 25% to 40% of patients [1–5]. However, pathophysiology of POAF is not fully disclosed [6–8]. General factors include older age, male gender, obesity, preexisting congestive heart failure, chronic renal failure, or COPD which are all risk factors for POAF [9–11]. Identification of patients who are at higher risk of POAF using genetic markers might be an important step for prevention of this type of operative complication and may provide important insight into the pathogenesis of atrial fibrillation (AF) and new therapeutic strategies for individual patients according to relative risk [12, 13].

During last 20 years, numerous AF associated mutations, candidate genes, and risk loci have been identified [14–19]; however, much of the heritability of AF remains unexplained [20]. Recently, Sinner et al [21] identified that 6 polymorphisms in 5 novel loci (NEURL rs12415501 and rs6584555, CAND2 rs4642101, GJA1 rs13216675, TBX5 rs10507248, and CUX2 rs6490029) were associated with atrial fibrillation susceptibility using a combination of genotyping, eQTL mapping, and functional validation. Are they also associated with POAF? To validate the hypothesis, we conducted this study to evaluated the positive findings for POAF susceptibility after CABG among Chinese population.

## RESULTS

### Clinical characteristics of the study participants

Characteristics of the cohort, stratified by the occurrence of POAF, are shown in Table 1. There were no significant differences between the POAF cases and the non-POAF cases for the mean age or gender distribution. This suggested that the matching based on these two variables was adequate. People were generally comparable for medical history of hypertension, diabetes, hyperlipidemia, and hemodialysis. While POAF patients have higher BMI (*P* = 0.005), and more likely to be COPD patients (*P* = 0.016), compared with the non POAF patients in stage I.

**Table 1:** Clinical characteristics of the study participants

	Stage I				Stage II	
Demographic	POAF (*N* = 600)	No POAF (*N* = 600)	*P* value	POAF (*N* = 800)	No POAF (*N* = 800)	*P* value
Age, y	65.5±5.2	65.8±5.9	0.350	62.7±4.9	62.9±4.3	0.386
Gender (% female)	152 (25.3%)	149 (24.9%)	0.842	204 (25.5%)	201 (25.1%)	0.863
BMI, kg/m 2	22.9±2.3	22.4±3.7	0.005	23.1±3.2	22.7±3.7	0.021
Medical history						
Hypertension	462 (77.0%)	455 (75.9%)	0.643	560 (70.0%)	567 (70.9%)	0.701
Diabetes	190 (31.7%)	161 (26.9%)	0.066	306 (38.2%)	295 (36.9%)	0.570
COPD	56 (9.3%)	34 (5.7%)	0.016	90 (11.2%)	86 (10.7%)	0.749
Hyperlipidemia	286 (47.7%)	269 (44.9%)	0.325	380 (47.5%)	366 (45.8%)	0.483
Hemodialysis	91 (15.2%)	79 (13.1%)	0.321	120 (15.0%)	110 (13.8%)	0.476

### Genotype distribution of 6 polymorphisms in 5 novel loci

Table 2 showed the genotypic frequencies of 6 polymorphisms in 5 novel loci (NEURL rs12415501 and rs6584555, CAND2 rs4642101, GJA1 rs13216675, TBX5 rs10507248, and CUX2 rs6490029) in POAF patients and non-POAF patients, together with their associations with POAF susceptibility after CABG among Chinese Han people. Four SNPs in 3 loci (NEURL rs12415501 and rs6584555, CAND2 rs4642101, and CUX2 rs6490029) were significantly associated with POAF susceptibility after CABG among Chinese Han people. For NEURL rs12415501, each additional copy of minor allele A was associated with a 1.29-fold increased risk of developing POAF (OR = 1.29, 95% CI: 1.03-1.60, *P* = 0.023) under the log-additive model. Compared with individuals with the GG genotype, the OR for developing POAF was 1.22 (95% CI: 0.92-1.63)among those with the AG genotype, and 1.51 (95% CI: 0.93-2.49) for those with the AA genotype. NEURL rs6584555 were also replicated in our study (per C allele: OR = 1.30, 95% CI: 1.06-1.60, *P* = 0.012). For CAND2 rs4642101, each additional copy of minor allele G was associated with a 1.30-fold increased risk of developing POAF (OR = 1.30, 95% CI: 1.06-1.55, *P* = 0.007) under the log-additive model. Compared with individuals with the TT genotype, the OR for developing POAF was 1.28 (95% CI: 1.02-1.67) among those with the TG genotype, and 1.57 (95% CI: 1.03-2.35) for those with the GG genotype. Another significant loci was CUX2 rs6490029, for which each additional copy of minor allele G was associated with a 0.77-fold decreased risk of developing POAF (OR = 0.77, 95% CI: 0.63-0.94, *P* = 0.008) under the log-additive model. Compared with individuals with the AA genotype, the OR for developing POAF was 0.88 (95% CI: 0.69-1.12) among those with the GA genotype, and 0.46 (95% CI: 0.28-0.76) for those with the GG genotype.

**Table 2:** Logistic regression analysis of genetic predictors of postoperative atrial fibrillation in the study populations of stage I

	POAF (*N* = 600)	No POAF (*N* = 600)	OR (95% CIs) *
NEURL rs12415501			
GG	420 (70.0%)	451 (75.1%)	1.00 (reference)
GA	139 (23.2%)	120 (20.0%)	1.22 (0.92-1.63)
AA	41 (6.8%)	29 (4.9%)	1.51 (0.93-2.49)
A vs G			1.29 (1.03-1.60)
P trend			**0.023**
NEURL rs6584555			
TT	424 (70.7%)	452 (75.3%)	1.00 (reference)
TC	105 (17.5%)	97 (16.1%)	1.15 (0.85-1.57)
CC	71 (11.8%)	51 (8.6%)	1.48 (1.01-2.17)
C vs T			1.30 (1.06-1.60)
P trend			**0.012**
CAND2 rs4642101			
TT	285 (47.5%)	329 (54.9%)	1.00 (reference)
TG	250 (41.7%)	223 (37.1%)	1.28 (1.02-1.67)
GG	65 (10.8%)	48 (8.0%)	1.57 (1.03-2.35)
G vs T			1.30 (1.06-1.55)
P trend			**0.007**
GJA1 rs13216675			
TT	200 (33.3%)	194 (32.3%)	1.00 (reference)
TC	260 (43.3%)	241 (40.1%)	1.05 (0.80-1.36)
CC	140 (23.4%)	165 (28.6%)	0.82 (0.61-1.11)
C vs T			0.90 (0.77-1.06)
P trend			0.204
TBX5 rs10507248			
GG	305 (50.8%)	293 (48.9%)	1.00 (reference)
GT	209 (34.9%)	211 (35.1%)	0.95 (0.74-1.22)
TT	86 (14.3%)	96 (26.0%)	0.86 (0.62-1.20)
T vs G			0.92 (0.78-1.09)
P trend			0.338
CUX2 rs6490029			
AA	375 (62.5%)	344 (57.3%)	1.00 (reference)
GA	202 (33.7%)	211 (35.1%)	0.88 (0.69-1.12)
GG	23 (3.8%)	46 (7.6%)	0.46 (0.28-0.76)
G vs A			0.77 (0.63-0.94)
P trend			**0.008**

* OR was adjusted by Age, gender, BMI and COPD

*P* value in bold means statistically significant.

### Replication of the 3 significant loci and functional validation

To enhance the reliability of the findings, we replicated NEURL rs6584555, CAND2 rs4642101, and CUX2 rs6490029 in an independent stage II. As shown in Table 3, NEURL rs6584555 and CAND2 rs4642101 were found to be significantly associated with POAF susceptibility. When pooled together, for NEURL rs6584555, each additional copy of minor allele C was associated with a 1.29-fold increased risk of developing POAF (OR = 1.29, 95% CI: 1.13-1.48, *P* = 1.7×10^−4^) under the log-additive model. Compared with individuals with the TT genotype, the OR for developing POAF was 1.46 (95% CI: 1.14-1.88)among those with the CC genotype; for CAND2 rs4642101, each additional copy of minor allele G was associated with a 1.21-fold increased risk of developing POAF (OR = 1.21, 95% CI: 1.08-1.36, *P* = 9.8×10^−4^) under the log-additive model. Compared with individuals with the TT genotype, the OR for developing POAF was 1.24 (95% CI: 1.06-1.45) among those with the TG genotype, and 1.38 (95% CI: 1.06-1.79) for those with the GG genotype.

**Figure 1:**
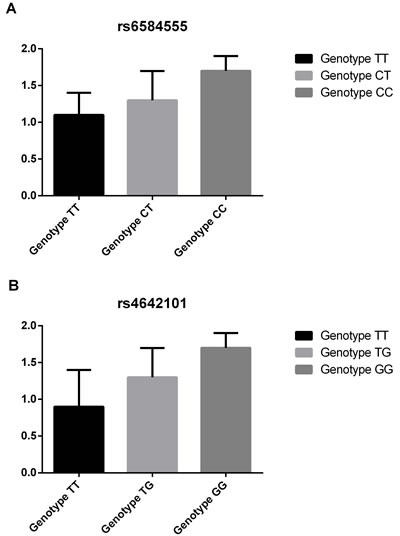
Associations between expression of NEURL, CAND2 and its genetic variants **A.** Associations between rs6584555 and expression of NEURL; **B.** Associations between rs4642101and expression of CAND2.

We then assessed the influence of NEURL rs6584555 and CAND2 rs4642101 on the expression of its corresponding genes in RAA samples. As shown in (Figure 1A and 1B), the AF risk allele of NEURL rs6584555 and CAND2 rs4642101 correlated with an increased expression of its corresponding genes (*P* < 0.001).

**Table 3:** Logistic regression analysis of genetic predictors of postoperative atrial fibrillation in the study populations of stage II

	Stage II			Pooled results		
	POAF (*N* = 800)	No POAF (*N* = 800)	OR (95% CIs) *	POAF (*N* = 1400)	No POAF (*N* = 1400)	OR (95% CIs) *
NEURL rs6584555						
TT	568 (71.0%)	605 (75.6%)	1.00 (reference)	992 (70.8%)	1057 (75.5%)	1.00 (reference)
TC	141 (17.6%)	128 (16.0%)	1.17 (0.90-1.53)	246 (17.6%)	225 (16.1%)	1.16 (0.95-1.42)
CC	91 (11.4%)	67 (8.4%)	1.44 (1.03-2.02)	162 (11.6%)	118 (8.4%)	1.46 (1.14-1.88)
C vs T			1.29 (1.07-1.54)			1.29 (1.13-1.48)
P trend			0.005			1.7×10^−4^
CAND2 rs4642101						
TT	394 (49.2%)	434 (54.2%)	1.00 (reference)	679 (48.6%)	763 (54.5%)	1.00 (reference)
TG	322 (40.2%)	293 (36.6%)	1.21 (0.98-1.49)	572 (40.8%)	516 (36.9%)	1.24 (1.06-1.45)
GG	84 (10.6%)	73 (9.2%)	1.26 (0.90-1.78)	149 (10.6%)	121 (8.6%)	1.38 (1.06-1.79)
G vs T			1.17 (1.00-1.36)			1.21 (1.08-1.36)
P trend			0.040			9.8×10^−4^
CUX2 rs6490029						
AA	472 (58.9%)	454 (56.8%)	1.00 (reference)			
GA	272 (34.0%)	280 (35.0%)	0.93 (0.76-1.15)			
GG	56 (7.1%)	66 (8.2%)	0.82 (0.56-1.19)			
G vs A			0.91 (0.77-1.07)			
P trend			0.252			

* OR was adjusted by Age, gender, BMI and COPD

P value in bold means statistically significant.

## DISCUSSION

Atrial fibrillation is a common arrhythmia with major public health implications due to its high prevalence, significant morbidity and considerable associated healthcare costs. There is strong evidence for heritability of POAF. In this large population-based, two-stage, nested case-control study, we identified 2 loci (NEURL rs6584555, and CAND2 rs4642101) were significantly associated with susceptibility of POAF after CABG. Testing for these genetic markers could improve risk stratification and potentially personalize therapy for preventing POAF.

The economic and clinical implications of postoperative AF are considerable. Many investigators have tried to identify predictors of AF after cardiac surgery for more than 2 decades. Although, the pathophysiology of AF has been extensively studied in the general population, it remains incompletely understood [22]. In 2003, Gaudino et al [23] identified that the −174 G/C polymorphism of the promoter of the Interleukin-6 gene has a role in the pathogenesis of postoperative atrial fibrillation. In current study, we identified that 2 loci (NEURL rs6584555, and CAND2 rs4642101) were significantly associated with increased risk of POAF after CABG. In the study of Sinner et al [21], the most significantly associated novel AF locus was also identified is intronic to the gene NEURL, which encodes an E3 ubiquitin ligase. The human NEURL has been recently determined and found to map to chromosome 10q25.1 within the region frequently deleted in malignant astrocytomas [24]. Using embryonic zebrafish, Sinner et al [21] found that knockdown of the NEURL orthologous specifically altered atrial action potential duration without affecting cardiac contractile function or heart rate. CAND2 gene encodes a TATA-binding protein, TIP120b, which is muscle-specific and critical for myogenesis, and the eQTL analyses also indicate that the risk allele is associated with increased expression of CAND2 [21]. Expression of CUX2 in post-mitotic neurons contributes to the maintenance of genome integrity through its stimulation of oxidative DNA damage repair [25]. Interesting, CUX2 loci were also significantly associated with ischemic stroke [21]. In our functional validation experiments, we found the AF risk allele of NEURL rs6584555 and CAND2 rs4642101 correlated with an increased expression of its corresponding genes (*P* < 0.001). These also proved the previous findings.

Our study has several limitations. We studied the genetic polymorphisms in Chinese undergoing CABG; therefore, our findings cannot be generalized to other ethnicities. In addition, we did not validate our findings in another independent cohort since the limited sample size we enrolled. We also didn't evaluated the interaction between these polymorphisms and preoperative BB, statin, anti-arrhythmic use, as well as their associations with term development of AF and mortality in CABG patients have not been previously described and will need further validation. Third, we used an α level of 0.05. Although the cohorts are likely underpowered for rigorous identification of associations by genome-wide genotyping techniques, surgical populations with detailed phenotype of AF or other adverse outcomes are rarely available. However, they examine important public health issues in an increasingly aged population.

Conclusively, NEURL rs6584555, and CAND2 rs4642101 are identified to be independently associated with risk of POAF. These findings delineate an important genetic role in the etiology of POAF and provide a detailed genomic landscape in which to examine biological mechanisms.

## MATERIALS AND METHODS

### Subjects

Patients undergoing primary CABG surgery without planned concurrent valve surgery were enrolled. The exclusion criteria were as follows: prior cardiac surgery; emergency surgical procedure; acute coronary syndrome; prior myocardial infarction; congestive heart failure; significant vascular heart disease; prior implantation of a permanent pacemaker, implantable cardioverter defibrillator, or cardiac resynchronization therapy defibrillator converted to a standard on-pump procedure; and use of class I or class III anti-arrhythmic agents. Also, the patients were excluded if they were not in sinus rhythm during echocardiography. POAF was defined as the occurrence of AF identified from nursing, physician, and perioperative ECG records, during the postoperative period of the primary hospitalization defined as the duration of contiguous hospitalization in the same institution as the surgery occurred. Finally, a total of 1,400 POAF patients and 1,400 non-POAF patients in two-stage were prospectively enrolled, respectively. An uniform questionnaire was used to collect patient demographics, preoperative and procedural factors, perioperative medication use, postoperative outcomes obtained from patient interview and medical records, and staff interviews. Right atrial appendage (RAA) samples of 200 individuals were randomly selected from the total patients with tissues available. The study protocols were approved by appropriate institutional review boards, and participants were enrolled after informed written consent was obtained.

### DNA extraction and genotyping

Genomic DNA was extracted from EDTA-anticoagulated peripheral blood leukocytes by the salting-out method [26]. The genotyping were determined by using a polymerase chain reaction-restriction fragment length polymorphism method (PCR-RFLP). To confirm the genotyping results, PCR-amplified DNA samples were examined by DNA sequencing, and the results were 100% concordant.

### Quantification of gene expression using real-time PCR

Expression of the candidate genes (NEURL and CAND2) in atrial tissues of 200 individuals who developed POAF was confirmed by real time PCR. POAF was present at the time of tissue acquisition Assays were performed using TaqMan gene expression probes and reagents (Life Technologies) and run on a 7900HT Real Time PCR System (Applied Biosystems). GAPDH was used as the reference gene.

### Statistical analyses

Genotype and allele frequencies of the SNPs were compared between POAF cases and controls using the x^2^ test and Fisher's exact test when appropriate, and odds ratios (OR) and 95% confidence intervals (CIs) were calculated to assess the relative risk conferred by a particular allele and genotype. For each of the SNPs, allelic associations with POAF were assessed using logistic regression analyses adjusted for the potential confounding factors. Demographic and clinical data between groups were compared by x^2^ test and by Student's-test. The linkage disequilibrium (LD) between the polymorphisms was quantified using the Shi's standardized coefficient D'. Statistical significance was assumed at the *P* < 0.05 level. The SPSS statistical software package version 11.5 was used for all of the statistical analysis.
